# Fiber Residual Stress Effects on Modal Gain Equalization of Few-Mode Fiber Amplifier

**DOI:** 10.3390/s23052574

**Published:** 2023-02-25

**Authors:** Li Pei, Yanbiao Chang, Jianshuai Wang, Jingjing Zheng, Tigang Ning, Jing Li, Bing Bai, Lei Shen, Li Zhong

**Affiliations:** 1 Key Laboratory of All Optical Network and Advanced Telecommunication Network, Ministry of Education, Institute of Lightwave Technology, Beijing Jiaotong University, Beijing 100044, China; 2 State Key Laboratory of Optical Fiber and Cable Manufacture Technology, Yangtze Optical Fibre and Cable Joint Stock Limited Company, Wuhan 430073, China

**Keywords:** modal gain equalization, few-mode fiber amplifier, fiber residual stress

## Abstract

The modal gain equalization (MGE) of few-mode fiber amplifiers (FMFAs) ensures the stability of signal transmission. MGE mainly relies on the multi-step refractive index (RI) and doping profile of few-mode erbium-doped fibers (FM-EDFs). However, complex RI and doping profiles lead to uncontrollable residual stress variations in fiber fabrication. Variable residual stress apparently affects MGE due to its impacts on the RI. So, this paper focuses on the residual stress effects on MGE. The residual stress distributions of passive and active FMFs were measured using a self-constructed residual stress test configuration. As the erbium doping concentration increased, the residual stress of the fiber core decreased, and the residual stress of the active fibers was two orders of magnitude lower than that of the passive fiber. Compared with the passive FMF and the FM-EDFs, the residual stress of the fiber core completely transformed from tensile stress to compressive stress. This transformation led to an obvious smooth RI curve variation. The measurement values were analyzed with FMFA theory, and the results show that the differential modal gain of the FMFA increased from 0.96 to 1.67 dB as the residual stress decreased from 4.86 to 0.01 MPa.

## 1. Introduction

Space division multiplexing (SDM) technology is considered a promising approach to break through the limit of single-mode fiber (SMF) communication system capacity [1,2]. As one of the implementation schemes of SDM technology, few-mode fiber amplifiers (FMFAs) are required to have a differential modal gain (DMG) between all amplified signal modes as small as possible, as a large DMG can increase the outage probability of the optical communication system [3,4].

Modal gain equalization (MGE) is closely related to the fiber refractive index (RI) and doping profiles of few-mode erbium-doped fibers (FM-EDFs). Conventional FM-EDFs with step refractive index (RI) profile and uniform doping always have high DMG (about 5–10 dB) [5,6], which cannot be used in communication systems. At present, in order to obtain an FMFA with a low DMG, various FM-EDFs with complex RI and doping profiles have been proposed and experimented with, such as those having ring-core RI profile and uniform doping [7], ring-core RI profile and layered doping [8], layered RI profile and layered doping [9], and air-hole-assisted layered/step RI profile and uniform/layered doping [10,11]. However, complex RI and doping profiles always lead to uncontrollable residual stress variations in fiber fabrication, which affects MGE due to residual stress being able to affect the fiber RI [12]. It is necessary to specifically study the effect of fiber residual stress on the MGE of FMFAs. The most basic way is to develop a configuration that can accurately measure fiber residual stress. At present, the scientific methods that can measure fiber residual stress include the half-shade method [13], the photoelastic tomography method [14], the two-wave-plate compensator method [15], and the Brace–Köhler compensator (BKC) method [16]. Among them, the BKC method is the most suitable technique for the actual measurement of fiber residual stress, because it can accurately measure lower optical retardation (caused by fibers) and its operation process is simple. Since its development, research on the effect of fiber residual stress on fiber performance has gradually developed. Raine et al. reported the axial stress profiles of UV-irradiated fibers, providing diagnostic information and the direct observation of UV-written grading structures [13]. In 2013, Feng et al. reported the effect of fiber residual stress changes caused by fiber cutting and arc fusion on the RI of large-mode-area (LMA) erbium-doped fibers (EDFs) and LMA ytterbium-doped fibers (YDFs) [17,18]. In 2016, Wang et al. reported the residual stress changes caused by weak arc discharge in single-mode fibers, and explored the influence of these changes on the filtering performance of long-period fiber grating [19]. In 2019, Anuszkiewicz et al. reported the effect of fiber residual stress of nanostructure-core fused silica fibers on the fiber RI and revealed that the residual stress of the fiber core was tensile stress [20]. In 2022, by measuring the residual stress in a single FBG fiber/epoxy composite system, Khadka et al. provided a new detection method for the fabrication of polymer systems and polymer matrix composites [21,22]. It can be found from the above research studies that fiber residual stress has an important influence on the performance of fiber systems.

In this work, the fiber residual stress effects on the MGE of FMFAs was studied. Using a self-constructed residual stress test configuration based on the BKC method, the residual stress distributions of passive and active few-mode fibers (FMFs) were measured. The increase in erbium doping concentration (EDC) caused a residual stress decrease in the fiber core, and the residual stress of the active fiber was two orders of magnitude lower than that of the passive fiber. Compared with the passive FMF and the FM-EDFs, the residual stress of the fiber core completely transformed from tensile stress to compressive stress. This transformation led to an obvious, smooth curve transition between the core layer RIs. The measurement values were analyzed with FMFA theory, and the results show that the DMG of the FMFA increased from 0.96 to 1.67 dB as the residual stress in the FM-EDF core decreased from 4.86 to 0.01 MPa, which reveals the relationship between residual stress and the MGE of FMFAs.

## 2. Principle

**Figure 1:**
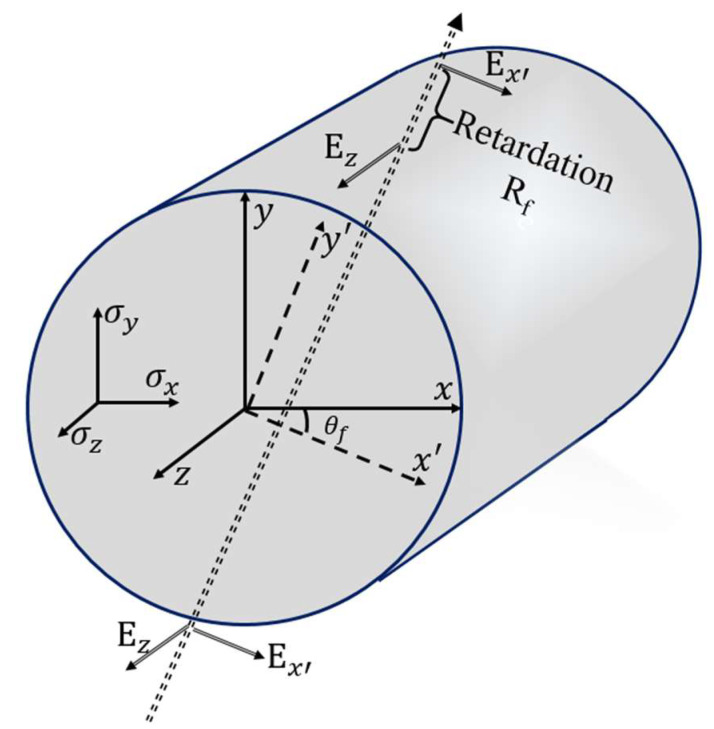
The diagram of optical retardation generated by the polarized beam passing through the fiber.

The residual stress test configuration was independently developed based on the BKC method, and the basic principle of the BKC method is analyzed and described in detail in reference [23]. The key point of this method is to measure the optical retardation generated by the polarized beam passing through the fiber, and the residual stress distribution of the fiber cross section can be calculated after the optical retardation distribution is processed using the inverse Abel transform [24] and computed tomography, as shown in Figure 1.

According to the stress-optic law, residual stress can change the fiber RI, and the specific expression is

(1) $$n=n_{0}+C\sigma$$

where *n*_0_ is the isotropic fiber RI; *C* is the elastic optical constant; *σ* is the residual stress, which can be decomposed into three stress components (*σ_x_*, *σ_y_*, and *σ_z_*); and *n* is the anisotropic fiber RI.

Next, we solve the fiber mode field distribution under the changed fiber RI, *n*. An optical fiber is a circularly symmetric regular optical waveguide, and its RI has invariance on the *z*-axis, that is,

(2) n(x,y,z)=n(x,y)

In the calculation of circular uniform optical waveguide, the optical vector satisfies the following wave equation:

(3) $$[\nabla^{2}+(k^{2}n^{2}-\beta^{2})]\left(E_{t}+E_{z}\right)=0$$

where ***E_t_*** and ***E_z_*** are the transverse and longitudinal components of the electric field, respectively. Moreover, ***E_t_*** also satisfies the wave equation and is expressed as

(4) $$[\nabla_{t}^{2}+(k^{2}n^{2}-\beta^{2})]E_{t}=0$$

In scalar field analysis, we adopt the rectangular coordinate system, and ***E_t_*** = ***E_x_*** + ***E_y_***, Since ***E_x_*** or ***E_y_*** in linear polarization mode is 0, let ***E_x_*** = 0 below. Then, by considering the circular symmetry of fiber waveguide and separating ***E_y_***,

(5) $$E_{y}(\rho ,\varphi )=e^{jm\varphi }E_{y}(\rho )e_{y}\;m=0,\pm 1,\pm 2,\cdots$$

Meanwhile, *E_y_(ρ)* satisfies the Bessel equation, i.e.,

(6) $$\frac{d^{2}E_{y}}{d\rho^{2}}+\frac{1}{\rho }\frac{dE_{y}}{d\rho }+\left(\frac{U_{}^{2}}{a^{2}}-\frac{m^{2}}{\rho^{2}}\right)E_{y}=0\;m=0,\pm 1,\pm 2,\cdots$$

where $U^{2}=\left(k^{2}n^{2}-\beta^{2}\right)a^{2}$. If $U^{2}<0$, $W^{2}=-U^{2}$. The solution of the above Bessel equation is

(7) $$E_{y}=\left\{ \begin{array}{l} AJ_{m}(U\rho /a)+BN_{m}(U\rho /a)\;kn>\beta \\ AI_{m}(U\rho /a)+BK_{m}(U\rho /a)\;kn<\beta \end{array}\right.$$

By solving the Maxwell equation, longitudinal field *E_z_* is obtained as follows:

(8) $$E_{z}=\left\{ \begin{array}{l} \frac{j}{\beta }\left[A\left(\sin\varphi \frac{U}{a}J\prime_{m}+jm\frac{\cos\varphi }{\rho }J_{m}\right)+B\left(\sin\varphi \frac{U}{a}N\prime_{m}+jm\frac{\cos\varphi }{\rho }N_{m}\right)\right]\;kn>\beta \\ \frac{j}{\beta }\left[A\left(\sin\varphi \frac{W}{a}I\prime_{m}+jm\frac{\cos\varphi }{\rho }I_{m}\right)+B\left(\sin\varphi \frac{W}{a}K\prime_{m}+jm\frac{\cos\varphi }{\rho }K_{m}\right)\right]\;kn<\beta \end{array}\right.$$

Equation (8) is the field distribution in the linear polarization mode. It can be seen that the fiber RI is influenced by residual stress, resulting in the change in the mode field distribution. According to the theory of FMFAs, the mode field distribution affects the gain performance of the amplifier. The gain performance of the FMFA is mainly related to the mode field distributions of signal light and pump light, and the doped particle distribution. It can be expressed as follows:

(9) $$\eta_{jk}=\iint_{S}Nt(\rho ,\varphi )i_{s,j}(\rho ,\varphi )i_{p,k}(\rho ,\varphi )\rho d\rho d\varphi$$

where *η_jk_* is the overlapping integral factor of signal mode *j*, pump mode *k*, and doped particle distribution *N_t_* on the cross section of the fiber, which determines the DMG; and *i_s,j_* and *i_p,k_* are the mode intensity distributions of signal light and pump light on the fiber cross section, respectively. It can be seen that as *N_t_* and *i_p,k_* are determined, *i_s,j_* is the only factor that can affect the DMG. Moreover, *i_s,j_* is closely related to the mode field distribution determined by refractive index *n*. Therefore, through the above calculations, the theoretical relationship between the fiber residual stress and the MGE of FMFAs is established. As for the solving of the DMG, it is analyzed specifically in our previous published work [9].

## 3. Experimental Measurement

**Figure 2:**
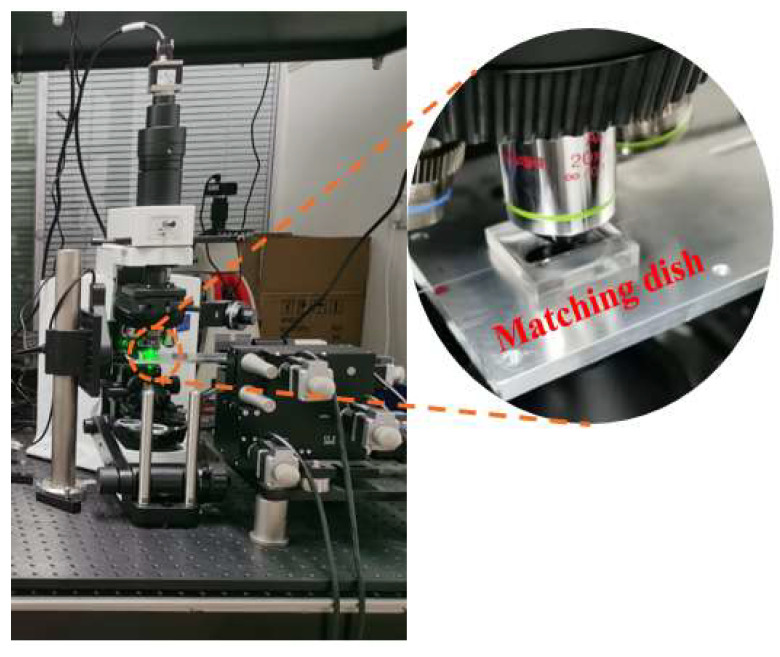
The self-constructed residual stress test configuration.

The experimental structure of the residual stress test configuration is shown in Figure 2. The fiber was placed in a glass concave matching dish with two micrometer holes on both sides, which prevented fiber offset. The glass concave matching dish is shown in the circular illustration in Figure 2.

**Figure 3:**
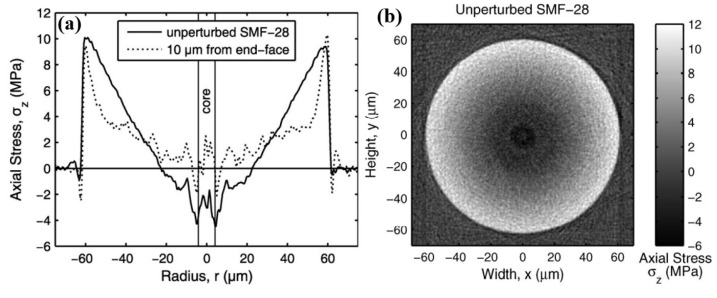
Reference residual stress distribution of Corning SMF−28: (**a**) one−dimensional distribution of axial residual stress; (**b**) two−dimensional distribution of residual stress in fiber cross section [16].

**Figure 4:**
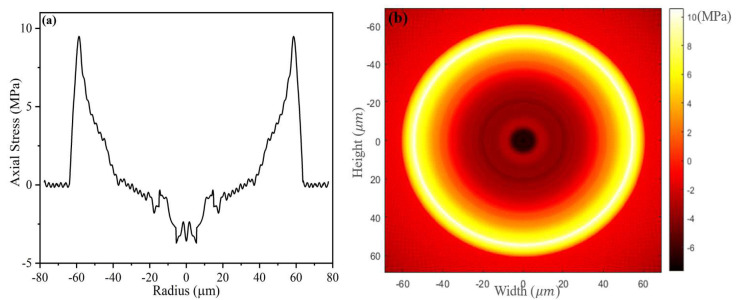
Actual measured residual stress distribution of Corning SMF-28: (**a**) one-dimensional distribution of axial residual stress; (**b**) two-dimensional distribution of residual stress in fiber cross section.

First of all, it was necessary to verify the measuring accuracy of the test configuration. The recognized method is comparing the residual stress measurement results of Corning single-mode fibers (SMF-28) with the published scientific work [16]. The representative references are the results published in Reference [16]. The residual stress distribution is shown in Figure 3. The measured residual stress distribution of Corning SMF-28 in this work is shown in Figure 4. Through comparison, the measured residual stress distribution of Corning SMF-28 in this work is similar to that of Reference [16], whether in one-dimensional or two-dimensional distributions. Moreover, at the outer edge of the fiber cladding, the residual stress reference value and the measured value are 9.74 and 9.65 MPa, respectively, showing a small difference. Therefore, according to the above verification, the measurement results of this residual stress test configuration are reliable.

Next, the test configuration was used to measure the residual stress of a series of home-made FMFs. These FMFs were produced according to the design of FM-EDFs in our previous work [9], including passive FMFs and FM-EDFs with different EDC. Here, different EDC refers to the amplification of the EDC ratio of each core layer [9] with different multiples to dope. Moreover, two kinds of FM-EDFs with low and high EDCs were successfully trial-produced. By measuring the residual stress distributions of these fibers, the influence of residual stress on the fiber RI was studied.

## 4. Result

### 4.1. Passive FMF

**Figure 5:**
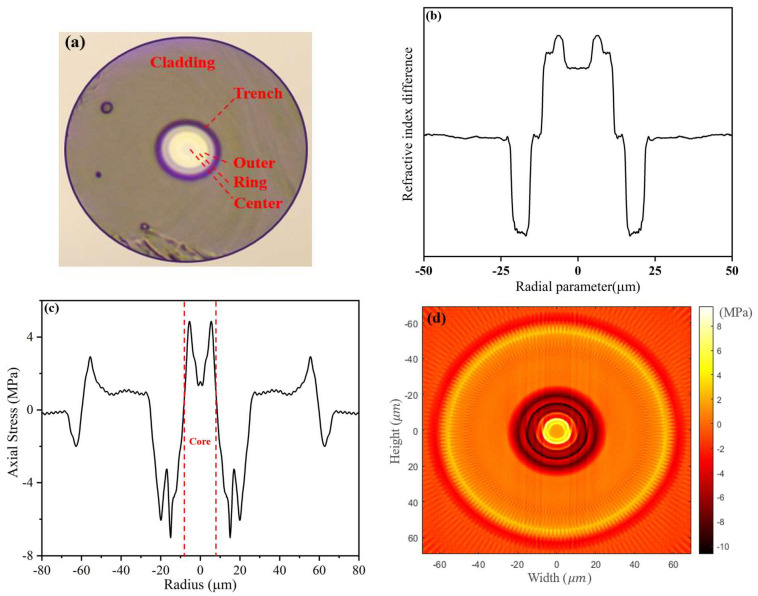
(**a**) Cross section, (**b**) RI profile, (**c**) and one-dimensional residual stress, and (**d**) two-dimensional residual stress distributions on the cross section of passive FMF.

The cross-section, RI profile, and one-dimensional and two-dimensional residual stress distributions of the passive FMF are shown in Figure 5. The passive FMF was a circular, single-cladding, trench-assisted, three-layered-core structure, as shown in Figure 5a. Figure 5b shows the RI profile, which was basically consistent with the design, and the RI transition of each core layer was broken. It can be seen in Figure 5c that the maximum residual stress value of the passive FMF core was 4.86 MPa around ±6.2 μm. Moreover, the residual stress in all core positions was greater than 0 MPa, indicating that the overall property of the FMF core residual stress was tensile stress (if the residual stress value is greater than 0 MPa, it is tensile stress; otherwise, it is compressive stress [23]). This tensile stress was mainly caused by the layered fiber core. In the process of the mechanical stretching of the FMF preform, the mutual adsorption and adhesion effects between multi-layered cores lead to tensile stress. As a comparison, that of the single-mode fiber core without layers was compressive stress, as shown in Figure 4a. Figure 5d shows the two-dimensional residual stress distribution on the cross section of the passive FMF.

### 4.2. FM-EDF-LAER

**Figure 6:**
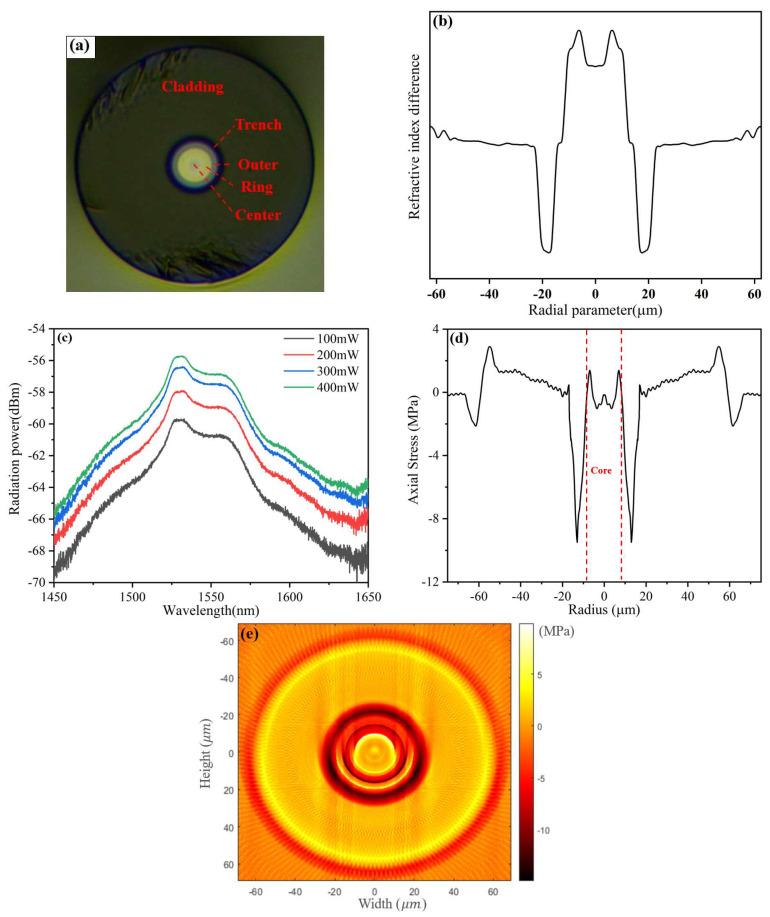
(**a**) Cross section, (**b**) RI profile, (**c**) spontaneous emission spectrum, and (**d**) one-dimensional residual stress and (**e**) two-dimensional residual stress distributions on the cross section of FM-EDF-LAER.

The cross-section, RI profile, spontaneous emission spectrum, and one-dimensional and two-dimensional residual stress distributions of the FM-EDF with a low-amplification erbium ratio (FM-EDF-LAER) are shown in Figure 6. The FM-EDF-LAER was a circular, single-cladding, trench-assisted, three-layered-core structure, as shown in Figure 6a. Figure 6b shows the RI profile; the RI flatness of each core layer decreased, showing a smooth curve transition trend between each core layer. In addition, in order to prove and distinguish the characteristic of the low-EDC FM-EDF-LAER, its spontaneous emission spectrum is shown in Figure 6c. When the selected FM-EDF-LAER length was 20 cm and the pump power was 400 mW, the spontaneous emission power (SEP) of the fiber in the wavelength range of 1528~1533 nm was about −55.76 dBm. Figure 6d,e show the one-dimensional and two-dimensional residual stress distributions of the FM-EDF-LAER, respectively. The maximum residual stress value of the FM-EDF-LAER core was 1.39 MPa around ±6.3 μm. The residual stress of the FM-EDF-LAER core showed a whole downward trend compared with that of the passive FMF core, and the residual stress in some core positions was less than 0 MPa, indicating that the residual stress in some core positions changed from tensile stress to compressive stress. This property change also conforms to the basic law of erbium particle doping in the core. Adding more components of particle materials to the limited core space causes the particles to squeeze each other, thus causing the residual stress in some core positions to decrease into compressive stress. This compressive stress led to the reduction in the RI flatness of some layers and to the transition of each layered core to show a smooth curve.

### 4.3. FM-EDF-HAER

**Figure 7:**
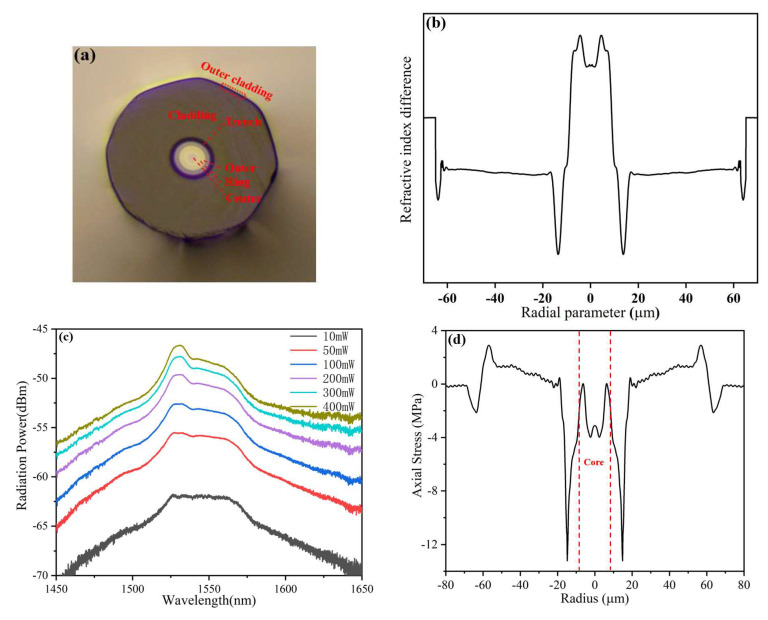

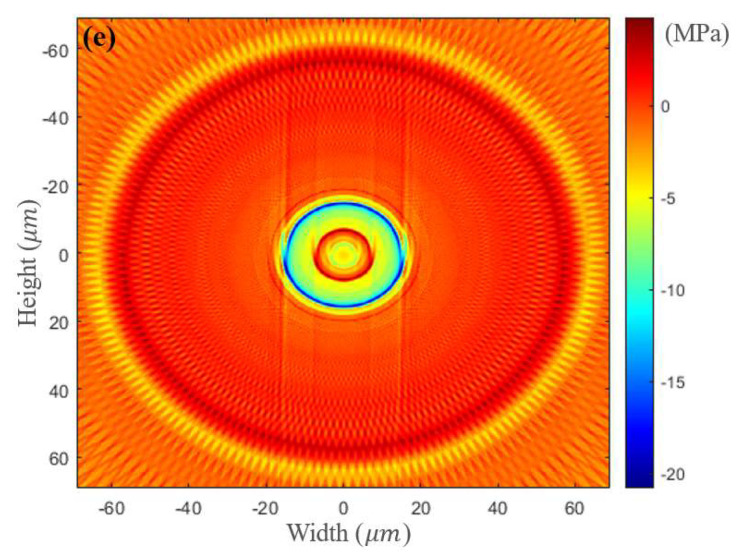
(**a**) Cross section, (**b**) RI profile, (**c**) spontaneous emission spectrum, and (**d**) one-dimensional residual stress and (**e**) two-dimensional residual stress distributions on the cross section of FM-EDF-HAER.

The cross-section, RI profile, spontaneous emission spectrum, and one-dimensional and two-dimensional residual stress distributions of the FM-EDF with a high-amplification erbium ratio (FM-EDF-HAER) are shown in Figure 7. The FM-EDF-HAER was an octagonal, double-cladding, trench-assisted, three-layered-core structure, as shown in Figure 7a. This octagonal outer cladding was conducive to the full utilization of pump light in the amplification experiment. Figure 7b shows the RI profile; the flatness of each core layer RI decreased more significantly, showing a smoother curve transition between each core layer than FM-EDF-LAER. The spontaneous emission spectrum of the FM-EDF-HAER is shown in Figure 7c. When the selected FM-EDF-HAER length was 20 cm and the pump power was 400 mW, the SEP of the fiber in the wavelength range of 1528~1533 nm was about −46.70 dBm, indicating that this fiber had a higher EDC than the FM-EDF-LAER. Figure 7d,e show the one-dimensional and two-dimensional residual stress distributions of the FM-EDF-HAER, respectively. The maximum residual stress value of the FM-EDF-HAER core was 0.01 MPa around ±6.3 μm. Compared with the case of the FM-EDF-LAER, the whole residual stress of the FM-EDF-HAER core was smaller and was less than 0.01 MPa, indicating that the residual stress in almost all positions of the FM-EDF-HAER core was compressive stress. The higher compressive stress made the materials of each core layer squeeze each other more significantly, resulting in lower flatness and smoother curve transition of the layered RI.

## 5. Discussion

**Figure 8:**
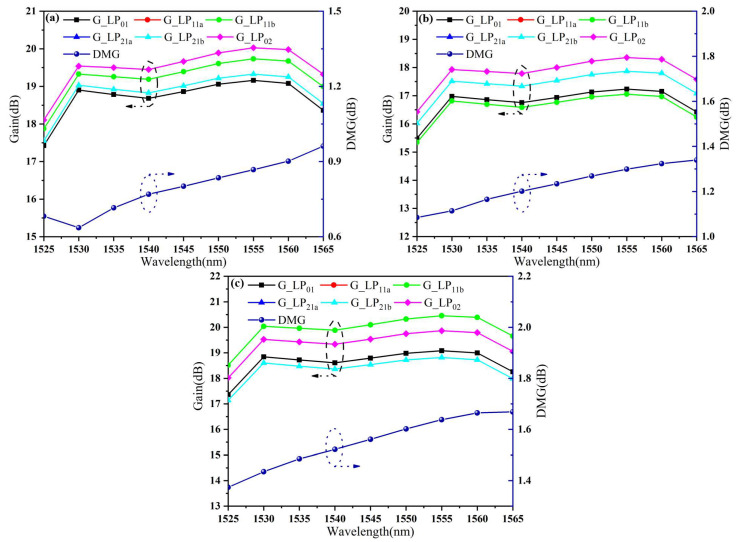
Modal gains and DMGs of (**a**) ideal fiber, (**b**) FM-EDF-LAER, and (**c**) FM-EDF-HAER as functions of the wavelength.

We analyzed the RI profiles of the above different fibers with FMFA theory [9] to investigate the MGE characteristics as follows:

(1) We assumed that there was an ideal FM-EDF-HAER, but with residual stress distribution and RI profile being consistent with those of the passive FMF, and marked it as the ideal fiber. In this case, the calculated modal gains and DMG as a function of wavelength are shown in Figure 8a. In the wavelength range of 1530~1565 nm, the DMG reached the maximum of 0.96 dB at 1565 nm, and the maximum DMG appeared between LP_02_ and LP_01_.
(2) The experimental RI profile of the FM-EDF-LAER was analyzed with FMFA theory to calculate the DMG. The corresponding results are shown in Figure 8b. Since the fiber had been doped with a lower EDC, the maximum signal modal gain was 18.35 dB, which was lower than the maximum modal gain (about 20.03 dB) of the ideal fiber. In the C-band range, the DMG reached the maximum of 1.34 dB at 1565 nm, and the maximum DMG appeared between LP_02_ and LP_11_.
(3) The experimental RI profile of the FM-EDF-HAER was analyzed with FMFA theory to calculate the DMG, and the corresponding results are shown in Figure 8c. The maximum signal modal gain of this fiber was 20.39 dB. Similarly, as the wavelength continued to increase, the DMG at 1565 nm reached a maximum of 1.67 dB. Moreover, the maximum DMG appeared between LP_11_ and LP_21_.

In conclusion, these residual stress changes not only affected the order arrangement of each modal gain in FM-EDFs but also led to a larger DMG, which was mainly caused by the influence of fiber residual stress on the RI. In this work, it was also found, for the first time, that with the increase in compressive stress, the transition of the layered RI showed a smooth curve trend, which had a significant impact on MGE. This discovery provides a new method for the reduction in MGE in the future and is conducive to the further improvement of communication system capacity. Finally, the influence of the above FM-EDF residual stress on the MGE of FMFAs is reported in Table 1. With the decrease in the core residual stress from 4.86 to 0.01 MPa, the DMG of the FMFA increased from 0.96 to 1.67 dB.

## 6. Conclusions

The presence and modification of residual stress inherently affects existing fibers and devices. In this work, the residual stress effects on the MGE of FMFAs was studied. Using a self-constructed residual stress test configuration based on the BKC method, the residual stress distributions of passive and active FMFs were measured. The results show that the increase in EDC caused the residual stress of the fiber core to decrease, and the residual stress of the active fibers was two orders of magnitude lower than that of the passive fiber. Compared with the passive FMF and the FM-EDFs, the residual stress of the core completely transformed from tensile stress to compressive stress. This transformation led to an obvious, smooth RI curve variation. Finally, the measurement values were analyzed with FMFA theory, and the results show that the DMG of the FMFA increased from 0.96 to 1.67 dB as the residual stress decreased from 4.86 to 0.01 MPa. This work is of great significance to the expansion of the research field of residual stress and reveals the relationship between residual stress and the MGE of FMFAs.

## Figures and Tables

**Table 1:** Influence of fiber residual stress on the MGE of FMFAs.

Fiber	Maximum SEP (dBm)	Peak Residual Stress in Core (MPa)	Property of Residual Stress in Core	RI Change	DMG (dB)
Ideal fiber	−46.70	4.86	Tensile stress	Broken layer transition	0.96
FM-EDF-LAER	−55.76	1.39	Partial transformation to compressive stress	Gradually smooth curve layer transition	1.34
FM-EDF-HAER	−46.70	0.01	Compressive stress	Totally smooth curve layer transition	1.67

## Data Availability

Not applicable.
